# A decline in the coverage and utilization of long-lasting insecticidal nets in Southern Ethiopia: A repeated cross-sectional study

**DOI:** 10.1371/journal.pone.0322342

**Published:** 2025-04-24

**Authors:** Misganu Endriyas, Tarekegn Solomon, Taye Gari, Teka Samuel, Bernt Lindtjørn

**Affiliations:** School of Public Health, Hawassa University, Hawassa, Sidama, Ethiopia; Freelance Consultant, Myanmar, MYANMAR

## Abstract

**Background:**

Despite ongoing interventions like long-lasting insecticidal nets (LLIN) distribution and indoor residual spraying, malaria is increasing in Ethiopia. LLIN ownership and utilization vary from time to time and place to place; thus, local evidence of LLIN ownership and use is required. Hence, this study aimed to investigate LLIN ownership, access within household, use, and associated factors using repeated indicators measures.

**Methods:**

A community-based cross-sectional study with repeated measures was conducted in the Sidama region, southern Ethiopia. The first survey was conducted in February and March 2023, and the second was done from October to December 2023. Multi-stage cluster sampling was employed to select representative households. LLIN coverage and use were estimated per the World Health Organization’s recommendations. Descriptive and multilevel logistic regression analyses were performed, and effect sizes were measured using adjusted odds ratios (AOR) with a 95% confidence interval (CI).

**Results:**

A total of 1647 households with 8054 individuals were included in the study. Most households were headed by males (89%), farmers (63%), and persons who were unable to read and write (55%). The ownership of at least one LLIN per household was 85% in survey one and dropped to 69% in survey two. The sufficiency of one LLIN for every two people was 36% in survey one and decreased to 29% in survey two. Similarly, the proportion of the population with access to LLIN within households decreased from 66.5% to 54.2%. Moreover, LLIN use dropped from 30.5% to 19.9% between the two surveys. The sufficiency of one LLIN to every two household members was consistently associated with geographical residence, sex, and education of household heads. In contrast, the education of household heads, family size, and age of individuals were consistent predictors of LLIN use. Females were likely to use LLIN during first survey, but no difference was noted during second survey.

**Conclusion:**

LLIN ownership and use were far below the conventional target (80%). LLIN ownership and utilization declined before the expected period. More than half of the population with access to LLIN within households do not use it. Thus, the malaria programs should consider more LLIN distribution and strengthen LLIN use among population with access to LLIN within their households.

## Introduction

Malaria is a major global public health problem, causing an estimated 249 million cases in 2022, and it disproportionally affects sub-Saharan Africa [1,2]. In 2022, Ethiopia contributed 2.1% of cases and 1.7% of global malaria deaths. Between 2021 and 2022, malaria incidence in Ethiopia increased by 32% (from 46.3 to 60.9 cases per 1000 population at risk) [1]. According to the Ministry of Health of Ethiopia, malaria is increasing in Ethiopia, and compared to cases in 2022, the number of cases doubled in 2023 [3].

Long-lasting insecticidal nets (LLINs) are among the primary vector control tools and can prevent malaria, even in the presence of pyrethroid resistance [2,4,5]. The World Health Organization (WHO) recommends LLINs for every person at risk of contracting malaria, which the Ethiopian National Malaria Guidelines adopt [6,7]. To ensure this recommendation, different progress-measuring indicators have been formulated. These include the proportion of households with at least one LLIN, those with one LLIN for every two people, the proportion of the population with access to LLIN within their household, and the proportion reporting having slept last night under LLIN [6,8].

The ownership of LLIN is measured in terms of possession of LLIN and whether available LLIN is sufficient for family size. Sufficiency of LLIN (the universal LLIN coverage or full household LLIN coverage) is defined as the household’s possession of one LLIN for every two people in the household. The proportion of the population with access to LLIN in their household indicates the proportion of the household population that could have slept under LLIN if two people had used each LLIN in the household. The proportion of the population reporting having slept last night under LLIN measures the level of LLIN use among all surveyed individuals [8].

Although every household member in malaria-endemic areas should sleep under LLINs every night, conventionally, the minimum population-based utilization that could provide sufficient benefit from LLIN has been set at 80% [9]. However, in sub-Saharan Africa, in 2021, the estimated percentage of the population with access to LLINs within their household was 54%, and the percentage of the population sleeping under LLINs was 47% [2].

In sub-Saharan Africa, studies indicated that LLIN ownership and utilization vary from time to time and place to place [10–12]. A systematic review and meta-analysis of LLIN use in Ethiopia also reported high variations in point prevalence of LLIN utilization, ranging from 14.2 to 91.9% [13]. Furthermore, cohort studies in Ethiopia demonstrated that LLINs last shorter than the expected three years due to high attrition and loss of integrity, resulting in low coverage and utilization [14,15]. These imply that LLIN ownership and use determined at one point may not be sufficient to inform malaria programs. However, most studies on LLIN use in Ethiopia used cross-sectional designs [13]. Thus, local evidence on LLIN ownership and use overtime is required.

Therefore, the overall aim of this study was to assess household malaria survey indicators using repeated measures of indicators. More specifically, the study aimed to determine (1) the ownership of at least one LLIN, (2) the sufficiency of one LLIN for every two people at household levels, (3) access to LLIN within the household, (4) utilization of LLIN at individual levels and (5) to compare differences in levels of indicators and risk factors between the surveys.

## Methods and materials

### Study setting

The study was conducted in the Boricha and Bilate Zuria districts of Sidama Regional State, southern Ethiopia. Boricha and Bilate Zuria are rural districts. Agriculture is the primary source of livelihood. In the study setting, districts are administratively subdivided into Kebeles (the lowest government administrative structure). Boricha has 14 *Kebeles,* while Bilate Zuria has 18 *Kebeles,* of which one *Kebele* is urban in each district. In 2023, Boricha had an estimated population of 132,397, and Bilate Zuria had 149,606. Boricha has one primary hospital, three health centers, and 13 health posts, while Bilate Zuria has five health centers and 17 health posts [16].

Boricha and Bilate Zuria districts are malaria-endemic districts in the region [17,18]. The study used the same *Kebeles* as previously described [19]. In Ethiopia, LLIN is distributed based on family size; one LLIN for a family with two members, two for three to four members, three for five to six members and the maximum number of LLIN provided for a family size of seven and above is limited to four [7]. In late 2022 (November and December), the government distributed LLIN through free mass campaigns in these districts. Although the study team observed the LLIN distribution at community level, the team had no role in LLIN distribution.

### Design and study period

Community-based, repeated cross-sectional surveys were conducted. The first survey was conducted in February and March 2023, while the second was done from October to December 2023. Data on LLIN ownership, LLIN use, and socio-economic characteristics of households and members were collected during the first survey. During the second survey, data on LLIN ownership and use were collected. This study was integrated with a malaria prevalence study.

### Study variables

The LLIN ownership and utilization indicators recommended by WHO and included in the Ethiopian National Malaria Guidelines were estimated in the present study [6,7]. These were ownership of at least one LLIN, the sufficiency of LLIN, access to LLIN within the household, and LLIN utilization.

The ownership of at least one LLIN was calculated by dividing households with at least one LLIN by the total number of households surveyed [8]. The sufficiency of LLIN was determined by dividing the number of households that met one LLIN for every two members by the total number of households surveyed. To determine whether a household met one LLIN for every two members (sufficiency of LLIN), the number of individuals who slept the previous night in the household was divided by the number of LLINs owned by the household. If the ratio was two or lower, the household met one LLIN for every two members [8].

The proportion of the population with access to LLIN in their household was determined by dividing potential users by the number of individuals who spent the previous night in surveyed households. The potential users were the number of individuals who could sleep under LLIN if two people used each LLIN in the household and calculated by multiplying the number of available LLINs by two. If the factor was greater than family size, it was corrected to be equal to family size [8]. The individual-level LLIN utilization was calculated by dividing the number of persons who slept under LLIN by the total number of people in the survey [6,8].

Data on the socio-demographic characteristics of all members in included households were collected. Educational status of individuals was assessed ordered category as cannot read and write, read and write, primary school, secondary school, and certificate and above. Age was collected in completed year. For LLIN utilization models, because of non-linear association, age was categorized per WHO’s suggestion and lowest category was set as reference [6]. In addition, assets such as house structures, agricultural assets (like the number of cattle and land size), and access to basic services like the availability of latrines and sources of drinking water were assessed.

### Population, sampling and sample size

Using a single population proportion formula in OpenEpi (online) and assuming the proportion of individuals using LLIN to be 50% (to estimate maximum sample size), a precision of 5% at 95% CI, the design effect of three and a non-response rate of 10%, the sample size became 1268. However, the sample size estimated for LLIN use was lower than the sample size estimated for the malaria prevalence study (1268 vs 1621 households). Thus, the sample size for the malaria prevalence study was used.

The sample size for the malaria prevalence survey was estimated using a single population formula considering an earlier estimate of malaria parasite prevalence measured by Rapid Diagnostic Tests (RDT). It was 1.17% among all age groups residing in 20 districts in five regional states and one city administration in Ethiopia [20]. Considering P = 1.17%, 95% confidence level, 0.3% absolute precision, and a design effect 1.5, the sample size was calculated to be 7367 persons. By adding 10% to account for the no response rate, the final sample size was estimated to be 8105 individuals. Considering a family size of five people per household, the sample size was 1621 households. By dividing the estimated sample size by 30, the cluster size was calculated to be 55.

A three-stage cluster sampling technique was used to select study participants for malaria prevalence surveys. The primary sampling unit was Kebele, the secondary sampling unit was *Limat Budin,* and the final was households*. The Limat Budin* is a local neighbor network of roughly 30 households. Proportional sample size allocation was done based on recent demographic data of households and populations from each kebele, which was based on population projections by the Central Statistical Agency [21]. Nine were selected from 30 rural Kebeles in two districts using a simple random sampling technique. There were 492 *Limat Budins* in the selected nine kebeles, and the required number of *Limat Budin* was selected from each kebele using a simple random sampling technique. All selected households and inhabitants in selected *Limat Budin* were included in the study. Residing for at least six months in *Limat Budin* was an inclusion criterion. Participants who refused to participate and/or were absent after three repeated visits were excluded.

**Fig 1:**
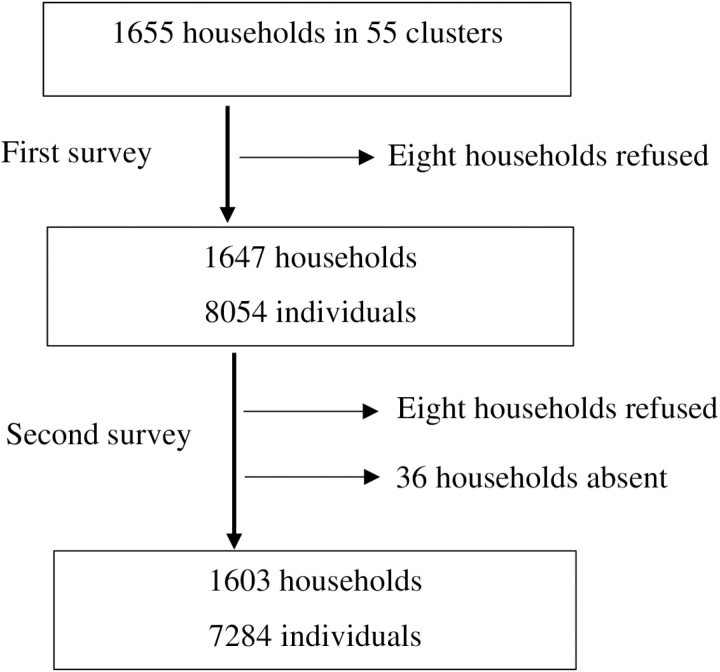
Flow-chart of the study.

There were 1655 households in selected clusters, of which eight refused participation during the first survey. There were 8054 individuals in the consented 1647 households. During the second survey, the same 1647 households included in the first survey were visited, of which another eight refused participation, and 36 were absent. Overall, 1603 households and 7284 individuals were included in the second survey (Fig 1).

### Data collection procedures

The data collection tools (S1 File) were adapted from the malaria questionnaire of Demographic and Health Surveys (DHS) version 8 [22]. A pair of experienced data collectors were assigned to each kebele and were overseen by two supervisors. The head of the household or another elder in the family of age 18 years or more was interviewed. All household members were registered, and the utilization of LLIN was assessed at household and individual levels. The ownership of LLIN was assessed by yes-or-no questions and verified by observation. The yes answers were followed by a question asking about the number of available LLINs. For each LLIN observed, utilization was asked. However, the hanging status of LLIN was not assessed since it could introduce bias. In the study setting, some people use spaces like living room as sleeping space and take off LLIN used in such places in the morining.

A native speaker translated the standard tool prepared in English to *Sidamu Afoo* and verified it by another native speaker. The interview was in *Sidamu Afoo*, the local language.

The data was collected by using Kobocollect on Samsung tablets. Data collectors and supervisors received three days of training on data collection tools. Investigators provided field supervision and onsite support to maintain data quality.

### Data management

Statistical analysis used Stata for Windows version 18 (StataCorp, College Station, TX, USA). All participants’ characteristics and outcomes were described using descriptive statistics. Using principal component analysis, a wealth index was constructed considering housing structure and assets [23]. Assets like TVs, cars, and refrigerators owned by less than five percent, and those (like land) owned by over 95 percent were excluded from the components. The 12 items used to construct the wealth index were ownership of a chair, bed, mobile, table, cemented floor, corrugated roof, cemented wall, pit latrine, bank account, separate kitchen, animal-driven cart, and any livestock (cow/ox/donkey/mule/sheep/goat). The Kaiser-Meyer-Olkin measure of sampling adequacy was 0.81. The first principal component had an Eigenvalue of 3.62 and a proportion of 30%.

The estimates of outcome variables during the surveys were compared using Pearson chi-square (for categorical variables), and a Wilcoxon signed-rank test was used to compare the number of LLINs available and the proportion of the population with access to LLIN in their household (because the distribution was non-linear). The LLIN utilization was calculated for subgroups of ages and sexes per the WHO recommendations [6].

Potential household-level predictors of LLIN sufficiency and household and individual-level predictors of LLIN use were tested using multilevel logistic regression. This was because multi-stage cluster sampling was used to select study participants, and the Intracluster Correlation Coefficient (ICC) showed significant differences at the cluster level for dependent variables, indicating the need for multilevel modeling [24].

For all regressions, independent predictors with a P-value of 0.20 or less in bivariable regression were considered in multivariable multilevel logistic regression to control confounding. In addition, residence (district), sex, age, and wealth index were included in the multivariable model as a known predictor. Multicollinearity among potential predictors was evaluated using the variance inflation factor (VIF). In addition to clusters included in the random effects model, variables supposed to vary between clusters, like wealth index, were tested both in fixed and random models. However, the addition of a random slope didn’t yield a significant difference in BIC. Hence, a fixed effect (random intercepts only) was reported. Finally, models with relatively lower AIC and BICs were selected and presented. Models were significant as compared to the null model (Wald Chi-square test) and logistic regression (likelihood-ratio (LR) test). The associations between dependent variables and predictors were declared at a P-value less than 5%, and the effect sizes were reported using an adjusted odds ratio with a 95% CI.

Ethical clearance was obtained from the Institutional Review Board, Hawassa University, College of Medicine and Health Sciences (Ref: IRB/087/14 and Date: 07/11/2022 and Ref: IRB/362/15 and Date: 29/07/2023). Support letter (Ref: DFI/7895/1 and Date 18/11/2022) was obtained from Sidama Region Public Health Institute and provided to lower structures following hierarchy. After explaining the study’s objectives, benefits, and risks, written informed consent was obtained from participants above 18 years, and assent for children under 18 was obtained from parents. Participants’ right to decline to participate was respected.

## Results

### Description of study participants

Of the surveyed 1647 households, 88.5% (1457 of 1647 household heads) were males, 71.8% (1182 of 1647 households) of household heads were farmers or housewives, and 55.2% (909 of 1647 households) of household heads were unable to read and write. Nearly half, 46.5% (3743 of 8054), of household members could not read and write. Tables 1 and 2 show the characteristics of households and individuals included in the first survey.

**Table 1:** Characteristics of households included in LLIN study, Sidama, 2023.

Variable	Categories	Frequency	Percent
Household characteristics (n=1647)			
District	Boricha	745	45.2
	Bilate Zuria	902	54.8
Family size	Less than five	737	44.3
	Five or more	910	55.3
Sex of head	Male	1457	88.5
	Female	190	11.5
Education of head	Can’t read and write	909	55.2
	Read and write	229	13.9
	Primary (1–8)	334	20.3
	Secondary (9–12)	117	7.1
	Certificate and above	58	3.5
Occupation of head	Farmer/Housewife	1182	71.8
	Trader	265	16.1
	Student	77	4.7
	Daily laborer	81	4.9
	Others	42	2.6
Age category of head	<=30	397	24.1
	31–40	476	28.9
	41–50	388	23.6
	51+	386	23.4
Marital status of head	Married	1490	90.5
	Widowed	131	8.0
	Others	26	1.6
Number of sleeping spaces	1	362	22.0
	2	905	54.9
	3	350	21.3
	4+	30	1.8
Wealth index	Lowest	310	18.82
	Second	347	21.07
	Middle	335	20.34
	Fourth	326	19.79
	Highest	329	19.98

**Table 2:** Characteristics of individuals included in LLIN study, Sidama, 2023.

Variable(n=8054)	Categories	Frequency	Percent
Sex of members	Male	4040	50.2
	Female	4014	49.8
Education of members	Can’t read and write	3743	46.5
	Read and write	428	5.3
	Primary (1–8)	2964	36.8
	Secondary (9–12)	694	8.6
	Certificate and above	225	2.8
Occupation of members	No job	1357	16.8
	Go to school	2685	33.3
	Farmer/Housewife	2572	31.9
	Trader	648	8.0
	Daily labor/housemaid	507	6.3
	Others	285	3.5
Age category of members	<5	710	8.8
	5–14	1999	24.8
	15–24	1865	23.2
	25+	3480	43.2
Marital status of members	Never married	4797	59.6
	Ever married	3257	40.4

### Ownership, sufficiency of and access to LLIN

The proportion of households having at least one LLIN was 85.3% (1405 of 1647 households) (95%CI: 83.5–86.9) during the first survey and was 69.2% (1109 of 1603 households) (95%CI: 66.9–71.4) during the second survey. The median number of LLIN owned by households during the first survey was 2 (interquartile range (IQR) = 1–2) and was higher than second survey, 1 (IQR = 0–1); Wilcoxon signed-rank test Z = -12.4, P<0.001.

The proportion of households that met one LLIN to every two family members was 35.8% (589 of 1647 households) (95%CI: 33.5–38.1) during the first survey and was 28.6% (459 of 1603 households) (95%CI: 26.5–30.9) during the second survey.

The proportion of the population with access to LLIN in their household was 66.5% (95%CI: 64.8–68.1) during the first survey and 54.2% (95%CI: 52.2–56.1) during the second survey.

### Predictors of the sufficiency of LLIN

After adjusting confounding and cluster effects during the first survey, households in Bilate Zuria were 86% less likely to have sufficient LLIN than households in Boricha. Compared to households led by males, households led by females were 2.66 times more likely to have sufficient LLIN. As the education of household heads increased by one level, the odds of having sufficient LLIN increased by 17% (Table 3).

**Table 3:** Predictors of sufficiency of one LLIN for every two people during first survey, Sidama, 2023.

Variable	Categories	Household met one LLIN to two members		Logistic regression				Multilevel logistic regression	
No n (%)	Yes n (%)	COR (95%CI)	P-value	AOR (95%CI)	P-value	AOR (95%CI)	P-value
Community level									
Districts	Boricha	347 (46.6)	398 (53.4)	1		1		1	
	Bilate Zuria	711 (78.8)	191 (21.2)	0.23 [0.19–0.29]	<0.001	0.21 [0.16–0.26]	<0.001	0.14 [0.07–0.26]	<0.001
Household level									
Sex of head	Male	954 (65.5)	503 (34.5)	1		1		1	
	Female	104 (54.7)	86 (45.3)	1.57 [1.16–2.13]	0.001	2.01 [1.43–2.83]	<0.001	2.66 [1.81–3.93]	<0.001
Age of head	Age of head	1058 (64.2)	589 (35.8)	0.99 [0.99–1.00]	0.27	0.99 [0.98–0.99]	0.02	0.99 [0.98–1.00]	0.11
Education	Education of head	1058 (64.2)	589 (35.8)	1.07 [0.98–1.16]	0.15	1.08 [0.97–1.20]	0.18	1.17 [1.03–1.33]	0.01
Occupation of head	Farmer/Housewife	772 (65.3)	410 (34.7)	1		1		1	
	Others	286 (61.5)	179 (38.5)	1.18 [0.94–1.47]	0.15	1.16 [0.89–1.51]	0.27	0.93 [0.69–1.25]	0.63
Wealth index	Wealth index	1058 (64.2)	589 (35.8)	1.00 [0.93–1.1.8]	0.92	0.92 [0.85–0.99]	0.03	0.95 [0.87–1.04]	0.26

### LLIN utilization

The utilization of at least one LLIN among households having LLIN was 77.9% (1095 of 1405 households) (95%CI: 75.7–80.0) during the first survey and was 53.8% (597 of 1109 households) (95%CI: 50.9–56.8) during the second survey.

During the first survey, the proportion of the population that slept under LLIN the previous night was only 30.5% (2458 of 8054 members) (95%CI: 29.5–31.5). During the second survey, this proportion declined to 19.9% (1447 of 7284 members) (95%CI: 19.0–20.8 (Table 4). A Wilcoxon signed-rank test showed the difference was significant (X2 = 228.7, P<0.001).

**Table 4:** LLIN use during first and second surveys, Sidama, 2023.

Variables	Number	Percent
LLIN use in survey one (n=8054)		
Yes	2458	30.5
No	5596	69.5
LLIN use in survey two (n=7284)		
Yes	1447	19.9
No	5837	80.1
LLIN use status in both surveys (n=7284)		
Used LLIN in both surveys	671	9.2
Did not use LLIN in either survey	4228	58.0
Did not use LLIN in the first survey but started in the second	776	10.7
Used LLIN in the first survey but stopped in the second	1609	22.1
Started LLIN use during the second survey among non-users during the first survey (n=5004)		
Yes	776	15.5
No	4228	84.5
Stopped LLIN use during the second survey among those who used LLIN during the first survey (n=2280)		
Yes	1609	70.6
No	671	29.4

**Fig 2:**
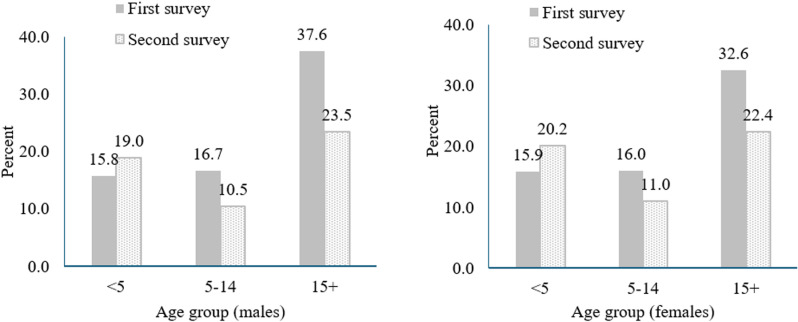
LLIN utilization disaggregated for age categories and sex, Sidama, 2023.

The LLIN utilization disaggregated for age and sex categories indicated that a higher proportion of age group 15 years or older were using LLIN than younger categories (Fig 2). Although a decline in the proportion of individuals using LLIN was noted for ages above five years, an increment in proportion was noted for children under five for both sexes. While LLIN use was similar for sexes over other age categories, a difference was noted for age categories 15 and above during the first survey (37.6% vs. 32.6%) (X^2^=480.5, P<0.001).

Of 7284 individuals with two-time measurements, only 9.2% (671 of 7284 individuals) (95%CI: 8.6–9.9%) used LLIN during both surveys, while 58.0% (4228 of 7284 individuals) (95%CI: 56.9–59.2%) did not use LLIN during both surveys. Additionally, of 5004 individuals who did not use LLIN during the first survey, 15.5% (776 of 5004 individuals) (95%CI: 14.5–16.5%) started using LLIN during the second survey. Moreover, of the 2280 individuals who utilized LLIN in the first survey, 70.6% (1609 of 2280 individuals) (95%CI: 68.7–72.4%) did not use LLIN during the second survey (Table 4).

### Predictors of LLIN use

After adjusting for confounding and cluster effects, the LLIN utilization was positively associated with the educational status of household heads and age of individuals and negatively associated with family size. More specifically, as the educational status of the household heads increased by one level, the odds of using LLIN among members increased by 32%. As the family size increased by one person, the odds of using LLIN among members decreased by 14%. Compared to males, females were 30% less likely to use LLIN. Moreover, compared to under-five children, individuals aged 15 years, and more were 4.82 times more likely to use LLIN (Table 5).

**Table 5:** Predictors of LLIN use among individuals during first survey, Sidama, 2023.

Variable	Category	Member used LLIN		Logistic regression				Multilevel logistic regression	
No No (%)	Yes No (%)	COR (95%CI)	P-value	AOR (95%CI)	P-value	AOR (95%CI)	P-value
Community level									
Districts	Boricha	2469 (69.9)	1064 (30.1)	1		1		1	
	Bilate Zuria	3127 (69.2)	1394 (30.8)	1.03 [0.94–1.14]	0.49	1.09 [0.98–1.21]	0.10	1.14 [0.72–1.83]	0.57
Household level									
Education	Education of head	5596 (69.5)	2458 (30.5)	1.25 [1.20–1.30]	<0.001	1.30 [1.24–1.36]	<0.001	1.32 [1.26–1.40]	<0.001
Occupation of head	Farmer/Housewife	4167 (70.3)	1759 (29.7)	1		1		1	
	Others	1429 (67.2)	699 (32.8)	1.16 [1.04–1.29]	0.01	0.98 [0.87–1.10]	0.74	0.92 [0.81–1.05]	0.22
Family size	Family size	5596 (69.5)	2458 (30.5)	0.85 [0.83–0.88]	<0.001	0.87 [0.85–0.90]	<0.001	0.86 [0.83–0.89]	<0.001
Wealth index	Wealth index	5596 (69.5)	2458 (30.5)	0.93 [0.90–0.96]	<0.001	0.94 [0.91–0.98]	0.001	0.99 [0.95–1.03]	0.58
Individual level									
Sex	Male	2667 (68.9)	1373 (31.1)	1		1		1	
	Female	2929 (75.5)	1085 (24.5)	0.72 [0.65–0.79]	<0.001	0.70 [0.63–0.77]	<0.001	0.70 [0.63–0.78]	<0.001
Age	< 5	598 (84.2)	112 (15.8)	1		1		1	
	5-14	1665 (83.3)	334 (16.7)	1.07 [0.85–1.35]	0.56	1.31 [1.03–1.66]	0.03	1.26 [0.98–1.62]	0.07
	15+	3333 (62.4)	2012 (37.6)	3.22 [2.61–3.97]	<0.001	4.11 [3.31–5.11]	<0.001	4.82 [3.84–6.07]	<0.001

### Comparison of indicators between surveys

The differences in estimates of the proportion of households with at least one LLIN, the proportion of households with one LLIN for every two people, the proportion of the population with access to LLIN within their households, and the proportion of individuals who slept under LLIN the previous night were statistically significant. For instance, the proportion of households with at least one LLIN for every two people decreased from 35.8% to 28.6% (P<0.001) (Table 6).

**Table 6:** Comparison of LLIN ownership and utilization at household and individual levels during two surveys, Sidama, 2023.

Variable	Category	Survey one		Survey two		Chi-square, P-value
n	%	n	%	
Households with at least one LLIN	Yes	1405	85.3	1109	69.2	120.6, <0.001
	No	242	14.7	494	30.8	
Households with at least one LLIN for every two people	Yes	589	35.8	459	28.6	18.9, <0.001
	No	1058	64.2	1144	71.4	
Proportion of population with access to LLIN in their household	^*^	^*^	66.5	^*^	54.2	Z = −11.9, P<0.001
Individuals slept under LLIN the previous night	Yes	2458	30.5	1447	19.9	228.7, <0.001
	No	5596	69.5	5837	80.1	

* Measured as continuous number for each household and difference was tested using Wilcoxon signed-rank test.

Regarding predictors, the sufficiency of one LLIN for every two household members was consistently associated with geographical residence, sex, and education of household heads during both surveys. However, confounding was noted in the case of the sex of the household head in both surveys (Table 3 and S5 File).

Age of individuals, educational status of household heads, and family size were associated with LLIN use during both surveys. The AORs of educational status of household heads and family size were similar. On the other hand, the females were less likely to use LLIN in the first survey, but no difference was noted in the second survey. In addition, compared to under-five children, children aged 5–14 were less likely to use LLIN in the second survey, but no difference was noted in the first survey (Table 4 and S5 File Models).

Lastly, family size and sex were associated with stopping LLIN use. As family size increased by one person, the odds of stopping LLIN use increased by 13%. Females were 1.63 times more likely to stop LLIN use (S5 File Models).

## Discussion

In this study, a rapid decline was observed in ownership of at least one LLIN, owning sufficient LLIN, access to LLIN within the household, and individual-level LLIN utilization within a short period. Moreover, the overall coverage and utilization of LLIN were low. The sufficiency of one LLIN to every two household members varied between geographical residence, sex, and education of household heads. In contrast, individuals’ age, household head education, and family size were consistent predictors of LLIN use. However, the sex of individuals was significantly associated with LLIN use in the first survey but not in the second survey.

To achieve the intended LLIN benefits, sufficient LLIN coverage is vital [9]. In the study setting, the government distributed LLIN based on family size, considering one LLIN for every two people in the household in November and December 2022, but limited the maximum LLIN to four for seven or more family members. The first survey conducted a month later, in February and March 2023, identified 85.3% of households owned at least one LLIN, a high number indicating recent distribution or reach of LLIN to the community. However, the sufficiency of LLIN was only 35.8%. This percentage declined to 28.8% during the second survey conducted from October to December 2023. This finding is far away from the 80% target.

Households in Bilate Zuria were 86% less likely to meet one LLIN for every two people. This could be due to limitations in the LLIN plan, or distribution or maintenance, which needs further investigation. Female-headed households were nearly threefold more likely to meet one LLIN for every two people. Data shows that female-headed households have smaller family sizes. This could be because most women in female-headed households in developing countries are widowed [25], thus may result smaller families. A study done in Tanzania also noted that female-headed households are small in size [26].

The educational status of household heads was positively associated with having one LLIN for every two household members and LLIN use among members. Other studies also indicated that education positively relates to LLIN ownership and use [14,27–29]. This might be attributed to the role of education in improving awareness. However, a study in Ethiopia demonstrated a contrasting association between schooling and LLIN ownership. Thus, it may be appropriate to ask if malaria prevention and control awareness might not result from formal education only but also health education [30].

An essential point for the malaria program is the gap between the proportion of the population with access to and the proportion of the population using LLIN. The gap was 66.5% access vs. 30.5% use during the first survey and 54.2% access vs. 19.9% use during the second survey, indicating more than half of the population with access to LLIN did not use it. This difference was larger than the sub-Saharan Africa estimate in 2021, which reported 54% access and 47% use [2]. This implies that along with availing LLINs, a lot has to be done on behavioral change and health promotion activities, as health education effectively increases LLIN use [31]. In addition, consistent use of LLIN is needed for malaria prevention. However, findigs of current study indicate as low as only 9.2% of individuals used LLIN during first and second surveys. The LLIN use was much lower than the pooled LLIN utilization in Ethiopia, which reported 56% [13], and the conventional 80% target.

As the number of family members increases, LLIN use among family members decreases. Cross-sectional studies in different African countries and a cohort study in Ethiopia indicated that larger families are less likely to use LLIN [14,32,33]. In Ethiopia, during LLIN distribution, the maximum number of LLIN provided for a family size of seven and above is limited to four [7]. In addition, the coverage maintenance distribution of LLIN is irregular. These could result in inadequate LLINs for households with large family size and, thus, low LLIN use among members.

Females were less likely to use LLIN in the first survey, but no difference was observed in the second survey. In addition, the sub-group analysis indicates that females are more likely to stop using LLIN from the first survey to the second survey. Different studies reported different associations of sex and LLIN use. Some studies from Africa, including a cohort study from Ethiopia, reported no association [14,32,33]. In some other studies, females were more likely to use LLIN [34,35], while others reported that females were less likely to use LLIN [36,37]. Although this can be attributed to socio-cultural practices, more contextual evidence is needed to understand the dynamics.

The LLIN use over categorized age groups indicates a higher proportion of under-five children use LLIN than school-aged children (5–14 years) during second survey, and the proportion was high for adults (15 and above) (Fig 2). Although the age categorization differs, the same pattern of association was noted in a study done in the Democratic Republic of Congo and Uganda [38,39]. A survey done in Madagascar reported that children under 5 years of age are prioritized for LLIN use because they are a known vulnerable age group for acquiring malaria, and in contrast, children over 5 years of age often sleep without LLINs because they are perceived to be at less risk for malaria [40]. This could also be due to an awareness of adults of the risks of malaria and LLIN use. While LLIN utilization decreased for school-aged children and adults during the second survey, it increased for under-five children. Although this might indicate that older LLINs are left for children, it needs further, perhaps, qualitative evidence to better understand the dynamics.

Our study included key indicators recommended by WHO, employed repeated measures and multilevel analysis. The consistencies in association between predictors and outcomes indicate validity of measures and association. However, its limitations that need to be improved were assessing the quality of LLIN and including some crucial predictors like knowledge and perceptions about LLIN. In addition, although LLIN ownership was verified by observation, the social desirability bias could have affected the measurement of LLIN use. To minimize this bias, houses were visited, preferably in the morning, the hanging of LLIN was observed, and interviewees were asked to report who slept under each LLIN by name. Moreover, even though repeated measures were taken, the levels of ownership and utilization were specific to the study period and should be interpreted cautiously. Since the study team had no role in LLIN distribution, we were limited in evaluating the quality of distribution and ensuring the reported universal coverage.

## Conclusions

Both LLIN ownership and utilization declined before the expected period. LLIN ownership and use were far below the expected levels to prevent and control malaria. The district, sex, and education of household heads were factors associated with the sufficiency of one LLIN for every two household members. LLIN use was associated with age of individuals, education of household heads, and family size. More than half of family members with access to LLIN within their family were not using LLIN. Malaria programs should consider more LLIN distribution and strengthen LLIN use among the population with access to LLIN within their households. Further qualitative evidence is required to understand the dynamics of LLIN use and gender variations within households.

## Supporting information

S1 File Questionnaire. Data collection tool in English and Sidamu Afoo.
(PDF)

S2 File HH data. Household data in long form.
(SAV)

S3 File Long members data. Members data in long form.
(SAV)

S4 File Wide members data. Members data in wide form.
(SAV)

S5 File Models. Models of second survey and additional variables.
(DOCX)
