# Supplementary material for: A decline in the coverage and utilization of long-lasting insecticidal nets in Southern Ethiopia: A repeated cross-sectional study
Source: PLoS One. 2025 Apr 24;20(4):e0322342. doi: 10.1371/journal.pone.0322342 (PMC12021140; doi:10.1371/journal.pone.0322342)
Supplement: S1 File — Data collection tool in English and Sidamu Afoo. (PDF) [file pone.0322342.s001.pdf]

Tool for LLIN utilization (English)

General Information		
**001**	Woreda	___________________________
**002**	Kebele	___________________________
**003**	**Limat budin**	___________________________
**004**	Household number	_______________________
**005**	Personnel (name and signature)	a) Interviewer____<br>b) Supervisor____
**006**	Date of visit	[_____|_____| ______| dd | mm| yyyy

Introduction and Consent

My name is____________ and I'm working for Hawassa University. In collaboration with the Woreda Health Office, we are conducting a survey on malaria. This information will help the Sidama Regional Health Bureau to plan health services. This interview could take less than 30 minutes to complete. Whatever information you provide will be kept strictly confidential and will not be shown to other persons. Participation in this survey is voluntary and you can choose not to answer any individual questions or all of the questions. However, we hope that you will participate fully in this survey since your views are important.

Do you have any questions about the survey? May I begin the interview now?

Written consent is given to interview, check box ☐

		Section 1: Household members' listing and socio-demographic and economic characteristics									
Q101	Total number of household members			Number ___________							
Start listing from the respondent him/herself											
Q102a		102b	102c	102d	102e	102f	102g	102h	102i	102j	102k
Individual ID	Household Members Name	Age	Sex	Relationship to head of household	Educational status	Marital Status	Current pregnancy status 1. Yes 2. No	Duration of pregnancy in months	Occupation	Ethnicity	Religion
1											
2											
3											
4											
5											
6											
7											
8											
9											
10											
11											
12											
13											
14											
15											

Sex

1. Male
2. Female

Relationship

1. head
2. Wife or husband
3. Child
4. Relative
5. Maid
6. Other

Educational Status

(6 years and above)

- **I=** Illiterate
- **RW=** Read and Write only
- **Elementary**
- **Secondary**
- **Certificate and above**

Marital Status (15 years & above)

1. Married
2. Living together
3. Divorced or separated
4. Widowed
5. Naver married/never lived together

Occupation

(18 years and above)

1. Employed
2. Housewife
3. Farmer
4. Day laborer
5. Trader
6. Student
7. No job/dependent
8. Housemaid
9. Others **(Specify__)**

**Q103**	Does your household have:<br>Electricity?<br>A watch?<br>A radio?<br>A television?<br>A mobile telephone?<br>A non-mobile telephone?<br>A refrigerator?<br>A table?<br>A chair?<br>A bed?<br>An electric mitad?<br>A kerosene lamp/pressure lamp?	Yes No<br>Electricity……………………………1 2<br>Watch…………………………………1 2<br>Radio…………………………………1 2<br>Television……………………………1 2<br>Mobile Telephone……………………1 2<br>Non-Mobile Telephone………………1 2<br>Refrigerator……………………………1 2<br>Table……………………………………1 2<br>Chair……………………………………1 2<br>Bed………………………………………1 2<br>Electric Mitad…………………………1 2<br>Kerosene/Pressure Lamp………………1 2	
**Q104**	Do you have a separate room which is used as a kitchen?	Yes…………………………………………1<br>No……………………………………………2	
**Q105**	Main material of the floor.<br><br>***(Record observation)***	Earth/Dung ……………………………...1<br>Ceramic Tiles…………………………….2<br>Cement………. . . . . ………….3<br>Other……………………………………96<br>Specify________________________	
**Q106**	Main material of the roof<br><br>***(Record observation)***	Thatch/Leaf………………………………1<br>Corrugated Iron . . . . ……... ………..2<br>Cement/Concrete . . . . . ……………... . . . . 3<br>Other……………………………………..96<br>(Specify)____________________	
**Q107**	Main material of the exterior wall.<br><br>***(Record observation)***	No wall……………………………………..1<br>Wood………………………………………..2<br>Wood with mud……………………………3<br>Wood with mud and cement………………..4<br>Cement blocks………………………………5<br>Other……………………………………..96<br>(Specify)____________________	
**Q108**	How many rooms in this household are used for sleeping?	Number of rooms[__|__]	
**Q109**	How many sleeping spaces such as mats, rugs, mattresses or beds are used in this household?		
**Q110**	Does any member of this household own:<br>A bicycle?<br>A motorcycle?<br>An animal-drawn cart?<br>A car or truck?	Yes No<br>Bicycle………………………………1 2<br>Motorcycle……………………………1 2<br>Animal-drawn cart……………………1 2<br>Car/truck………………………………1 2	
**Q111**	Does any member of this household own any land that can be used for agriculture?	Yes…………………………………………1<br>No……………………………………………2	**Skip to Q113**
**Q112**	How many (LOCAL UNITS) of agricultural land do members of this household own?<br>***(If unknown enter 98)***	Local units [___|___]<br>Specify the local unit__________________	
**Q113**	Does this household own any livestock, herds, or farm animals?	Yes…………………………………………1<br>No……………………………………………2	
**Q114**	How many of the following animals does this household own?<br>Milk cows, oxen, or bulls?<br>Horses, donkeys, or mules?<br>Goats?<br>Sheep?	Milk cows, oxen, or bulls------ [| |]<br>Horses, donkeys, or mules----- [| |]<br>Goats----------------------------- [| |]<br>Sheep----------------------------- [| |]<br>Chickens------------------------- [| |]	

	Chickens?<br>***(If unknown, enter 98)***		
**Q115**	Does any member of this household have an account with a bank/credit association/micro finance?	Yes……………………………………….1<br>No……………………………………….2	
**Q116**	What is the main source of drinking water for members of your household?<br><br>***(Do not read out Responses)***	**Piped (Tap)**<br>Piped into dwelling……..…1<br>Piped into compound……...2<br>Piped outside compound….3<br>Covered Well…………..…4<br>Protected Spring………..….5<br><br>**Open Well/Spring**<br>Open Well…………………6<br>Open Spring……………..…7<br><br>**Surface Water**<br>River……………….………..8<br>Pond/Lake/Dam…..………..9<br>Rainwater………………10<br><br>Other…..……….………..11<br>Specify______________________	
**Q117**	What kind of toilet facility do most members of your household use?<br><br>***(observe latrine)***	Flush toilet…………………..…...………..1<br>Pit latrine/traditional pit toilet..……….2<br>Ventilated improved pit latrine (VIP) …3<br>No facility/Bush/Field… ………….….4<br>Other………….…………………….…5<br>Other(Specify)______________________	→ **Skip to Q201**
**Q118**	Do you share this facility with other households?	Yes…………….1<br>No..…………...2	

**Section 2: Malaria prevention and treatment**					
**Q201**	Does your household have any mosquito net that can be used while sleeping?	Yes…………….1<br>No..…………..2 →			**Skip to Q211**
**Q202**	How many mosquito nets do your household have?	Number of Nets _________			
**Q203**	Ask respondent to show you the net(s) in the household.	NET #1 ______<br>Observed .... 1<br>Not observed......... 2	NET #2 _______<br>Observed..............1<br>Not observed 2	NET #3 _______<br>Observed................1<br>Not observed.. 2	
**Q204**	How long ago did your household obtain the mosquito net?	____ ____ Months ago	____ ___<br>Months ago	____ ____<br>Months ago	
**Q205**	Where did you obtain the net?	Government Clinic/hospital Health extension worker……………….1	Government Clinic/hospital Health extension worker…………….1	Government Clinic/hospital Health extension worker…………….1	

		Retail shop<br>Pharmacy…………….2<br>Workplace……………3<br>Other<br>(specify)__________.....4<br>Don't know................98	Retail shop<br>Pharmacy………….2<br>Workplace………..3<br>Other<br>(specify)_________....4<br>Don't know……….98	Retail shop<br>Pharmacy………….2<br>Workplace………..3<br>Other (specify.........4<br>Don't know…….98	
**Q206**	Did you purchase the net?	YES............1<br>NO...............2<br>Not sure............ 8	YES............1<br>NO..........2<br>Not sure............ 8	YES............1<br>NO.............2<br>Not sure............ 8	**skip to 208**
**Q207**	How much did you pay for the net when it was purchased?	_________ birr	_________ birr	_________ birr	
**Q208**	Did anyone sleep under the mosquito net last night?	Yes…………….1<br>No..…………...2<br>Not sure………8	Yes…………….1<br>No..…………...2<br>Not sure………8	Yes…………….1<br>No..…………...2<br>Not sure………8	**Skip to Q210**
**Q209**	Who slept under this mosquito net last night?	Individual ID<br>1. __________<br>2. __________<br>3. _________<br>4. ________	Individual ID<br>1. __________<br>2. __________<br>3. _________<br>4. _________	Individual ID<br>1__________<br>2__________<br>3_________<br>4_________	
**Q210**	Why did no one sleep under this mosquito net last night?	No malaria………….. 1<br>No nuisance/insects… 2<br>No space for net ……….3<br>Irritation ………………4<br>Suffocation / too hot ….5<br>Difficult hanging net ….6<br>Shape ………………..7<br>Absence from home …..8<br>Other.......................... 9<br>Don't know……....98	No malaria………. 1<br>No nuisance/insects.. 2<br>No space for net ….3<br>Irritation …………4<br>Suffocation / too hot ..5<br>Difficult hanging net ..6<br>Shape ………….7<br>Absence from home ..8<br>Other......................... 9<br>Don't know……….98	No malaria….. 1<br>No nuisance/insects.. 2<br>No space for a net …3<br>Irritation …………4<br>Suffocation / too hot ...5<br>Difficult hanging net ..6<br>Shape ……………..7<br>Absence from home…..8<br>Other.......................... 9<br>Don't know……….98	
**Q211**	Was your house ever been sprayed with insecticide for malaria prevention by spraymen from the District Health Office?	Yes…………….1<br>No..…………...2<br>Not sure..……….8			**Skip to Q215**
**Q212**	How many months ago was your house sprayed?<br>***(If less than one month, record 0)***	Months ago [___/___]<br>Not sure…..8			
**Q213**	At any time in the past 12 months, have the walls in your dwelling been plastered or painted?	Yes.. ...1<br>No... 2			
**Q214**	How many months ago were the walls plastered or painted? *If less than one month, record 0.*	MONTHS AGO, _____ _____			

Tool for LLIN utilization (Sidamu Afoo)

**Xaphoomu Taje**		
**001**	**Woreda**	________________
**002**	**Kebele**	________________
**003**	Fayyimmate olanto gaamo	________________
**004**	Mini maate kiirotenni	________________
**005**	Xa'maanchunna qorqoraanchu su'ma :malaate	a) Xa'manchu su'manna malaate______________<br>b) Qorqqoraanchu su'manna malaate___________
**006**	Towanyote barra	[_____|_____| ______| barra | agana<br>| diro

Bitimanna Fajjo

Su'ma'ya ____________ yinannie. Ani looseemmohu Hawaasi yuniveristeraati. Ninke assinemo laooshshi mini-wiliilchunna woradu fayyimmate biiro waaxo xiinxallateeti. Uytinanninke taje Sidaaamu Dagoomu Qoqqowu Mootimma fayyimmate mixo owaantera fakkana kaa'lo uytanno. Tini qaali-xa'mo keeshshitannohu 30 xiqqeessaati. Uytinanninke taje fojo wolu mannira dihasaambanni/ dikullanni. Beeqqo assattohuno/ahuno meessikki fajjonniiti. Ikkollana, laooshshikki dancha ikkiro, beeqqo asssatto/a gede hexxo'yaati. Aanino diri bocu-boci diri giddo towaanyo heedhanno.

Tenne xiinxallora xa'mo noohe? Xa'mo hanafo

Borrote fajjo anga'nera oommo ☐

		Gafa 1: Mini Maate Su'mi Tittironna Dagoomi-Miinju Akata									
X101	Xaphoomunni Mini Maate Kiiro	kiiro __________									
Minu anniwiinni woy amawiinni hanaffe antentenni maate borreessi											
X102	A	102b	102c	102d	102e	102f	102g	102h	102i	102j	102k
Ayim mate Kaarde	Mini maate su'ma	Diro	Koo/ tee	Minu anni ledo no fixoo ma	Rosu deerra	Adh aa-mate dana	Xaa yannara godowinni noohuno? 1. Ee 2. Dee'ni	Godowinta-nkunni me''e agana ati?	Loosu dana	Hiikko ayiddeeti	Ama'no kki?
1											
2											
3											
4											
5											
6											
7											
8											
9											
10											
11											
12											
13											
14											
15											

Koo/Tee

1. koo
2. Tee

Fiixooma

1. anna/ama
2. Galte/gashshaanna
3. Qaaqqo
4. fiixa
5. soqqantannota
6. wole

Rosu derra

(6dirihuranna aliidihura

- **I=**Rosinokkiho
- **RW=** Nabbawanna borreessa dandiinoho
- ***U*mi dirimira rossanno**
- **Layinki dirimira rossanno**
- **Sertifikeettetenna hakkuy ale**

Adhamate dana 15 diri alihura calla

1. Adhaminoho
2. Mitteenni he'ranno
3. Tidhaminoho woy baxxinoho
4. Shiidhinote
5. Adhaminokkiho/mitteenni hee'ranokkiho

Loosu dana

*(**18** tehonna aliidihura)*

1. Qaxaraminoha
2. Minu ama
3 Baatto loosi're galinoha
4. Barru loosaasincho
5. Daddalaancho
6. Rosaanchoho
7. No job/dependent
8. Housemaid
9. Others (Specify__)

Q103	Mini'ne maatera no?<br>Korreente<br>Girgiddu saate<br>Raadoone<br>Televizhiine<br>Mobayile(kiisete bilbili)<br>Mini bilbili<br>Qiissancho?<br>Xarapheezzu?<br>Barcimu ?<br>Daallasu?<br>Korreentete loossanno mixashsho?<br>Kurraaze/maasho	1 Ee 2 Dee'ni<br>Korreente---------------------1 2<br>Girgiddu saate --------------1 2<br>Raadoone------------------- 1 2<br>Televizhiine ----------------- 1 2<br>Mobayile(kiisette bilbili)----1 2<br>Mini bilbili--------------------1 2<br>Qiissaancho?------------------1 2<br>Xarapheezzu? ----------------1 2<br>barcima -----------------------1 2<br>Daallasu? ---------------------1 2<br>Korreentete loossanno mixashsho 1 2<br>Kurraaze -----------------------1 2	
Q104	Sagale qishi'nanni addi kifile noo'ne?	Ee……………1<br>Dee'ni…………….2	
Q105	Mini uullaydo mayinni loonsoonni?<br><br>*(Laoottore wonshi)*	Bushshunni/obbunni…………...1<br>shakilunni……..……… ……..…..2<br>Simintote……….. . . . ……... . . .3<br>wole…………………………….96<br>_	
Q106	Mini fukko mayyini loonsonni?<br><br>*(Laoottore wonshi)*	hayisso/darote………………….……1<br>Qorqorote. . . . . ……....……..2<br>simintote. . . . . ……………... . . . . 3<br>wole………….……………………..96<br>(xawisi)____________________	
Q107	Mineho gobbaydi girgidda mayinni loonsoonni?<br><br>*(Laoottore wonshi)*	Girgiddu dinooho………………..1<br>Haqqeete………………………….2<br>Haqqetenna sabbunni…………….3<br>Haqqetenni,sabbunninna simintotenni………………………4<br>Bilokeettete………………<br>Wole ……………………………..96<br>(xawisi)_____________________	
Q108	Me''e kifile noo'ne gonxanniti mini'ne maatera ?	Kifile kiirotenni wori[__|__]	
Q109	Meu gonxanni baychi noo'ne daddo,madawe/shara /daallassa kadhate gonxanni baychi noo'ne?		
Q110	Maate'ne giddo kuriuu noohu no?:<br>Shalleette?<br>xexxersa?<br>Gaare…?<br>Kaameella?	Ee Dee'ni<br>shalleette…………………….1 2<br>xexxersa…………..………1 2<br>Gaare……………...……...…1 2<br>Kaameella……………………1 2	
Q111	Maate'nera baatto loossidhinanniti noo'ne?	Ee…………1<br>Dee'ni…….2 →	Sai xa'mo X113

Q112	Maate'ne loosidhe hedhanno baatto me''ete?Qarqaru kiironni?<br>*(anfoonnikkiha ikkiro 98 kiiro borreessi.*	Qarqaru bikkinni [___|___]<br>xawisi__________________	
Q113	Mini'ne maatera lalu,hoshsha/ce'inoonni saada no?	Ee…………1<br>Dee'ni…….2	
Q114	Maate'nera me''e saada noo'ne umi'neti?<br>Adote saada, handa, /aja bootta farado, harre, gaango?<br><br>Meu?<br>Ge'reewo?<br>lukkuwa?<br>*(anfoonnikki ikkiro 98 kiiro borreessi)*	Adote saada, haanda, /aja bootta------<br>farado, harre, gaango----<br>Meu------------------------------<br>Ge'reewo------------------------------<br>lukkuwa--------------------------	
Q115	Maate'ne giddo woxe suuqidhinanni baankete/maykiro fayinanse kiiro noo'ne?	Ee………1<br>Dee'ni……….2	
Q116	Mini'ne maate duucha wote agate horoonsidhinanni way hiikkiinniiti?<br><br>*(Dawaro horonta nabbabbooti)*	Meessiha baambu waa xorshi'rate …1<br>Hoowenke giddo noo baamba horoonsi'neemmo…2<br>Hooenke gobbaanni nooha baambu waa ….3<br>Tuantino balenni …………..…4<br>Huxxamino burqanno waa ……5<br><br>Fano bale /burqanno waa……………6<br>Fano buqanno waa …7<br><br>Daadanno waa<br>Lagga ……………..8<br>xashsho/garba /kofatto….….9<br>xeenu waa……….10<br><br>wole xawisi …..……….11<br>______________________	
Q117	Mini'ne maate shumate horoonsidhanno mini hiittooho?<br><br>*(laoottoha shumate mine borreessi)*	Shumate horoonsi'rate injaanno mine .1<br>Sammi yine ummoonni shumate<br>bale/bashsho rosamino shumate mnie ..2<br>Silancho yannicha shumate mine …3<br>Takkontanni dino/ /citu giddo/mullawa<br>xawo… …….4<br>Wole xawisi ………….…………5<br>______________________	→ X201 sai

Q118	Konne shumate mini'ne gutunni horoonsidhinanni?		Ee …………….1<br>Dee'ni ……..2		
Gafa 2: Shekkeere Gargadhanna Xagisa					
Q201	Mini'ne maate giddo 'agoberre' goxanno wote horoonsi'rannohu no ?		Ee …………….1<br>Dee'ni ……………2		X 211 Sai
	Mini'ne maate giddo me''e agaoberre noo'ne ?		Kiirotenni _________		
Q203	Xa'mamaancho mini'ninsa giddo agoberre nooro xa'mi	| #1ki |agobere ______	#2ki agobere _______	#3ki agobere _______	
		Laoommo .... 1<br>Dilaoommo 2	Laoommo .... 1<br>Dilaoommo 2	Laoommo .... 1<br>Dilaoommo 2	
Q204	Maatekki Konni albaanni agoberre mageeshshi yanna afidhino?	____ ____ agannate albaanni	____ ___<br>Agannate albaanni	____ ____<br>Agannate albaanni	
Q205	Agoberre maminni afidhinanni?	Mootimmatewiinni Kilinikete /hospitaalete Fayymmate ekistenshinete loosaasinewiinni ………………1<br>Suuqetenni Xagichu mininni ……………2<br>Loosu darginni ……………3<br>Wole xawisi.....4<br>Diafoommo/a yiiro/turo…98 kiiro borreessi	Mootimmatewiinni Kilinikete /hospitaalete Fayymmate ekistenshinete loosaasinewiinni ………………1<br>Suuqetenni Xagichu mininni ……………2<br>Loosu darginni ……………3<br>Wole xawisi.....4<br>Diafoommo/a yiiro/turo…98 kiiro borreessi	Mootimmatewiinni Kilinikete /hospitaalete Fayymmate ekistenshinete loosaasinewiinni ………………1<br>Suuqetenni Xagichu mininni ……………2<br>Loosu darginni ……………3<br>Wole xawisi.....4<br>Diafoommo/a yiiro/turo…98 kiiro borreessi	
Q206	Agobere hidhitine horoonsidhin-anni?	Ee ....1<br>Dee'ni ....2<br>Dibuuxoomma/o........... 8	Ee ....1<br>Dee'ni ....2<br>Dibuuxoomma/o............ 8	Ee ....1<br>Dee'ni ....2<br>Dibuuxoomma/o............ 8	►sai X 208
Q207	Agoberete mageeshshi baatooshshe baatte hidhitta/o?	_________ Birrinni	________ birrinni	_________ birrinnni	
Q208	Maate'ne giddo , ankarro hashsha agobere horoonsi'rinohu no ?	Ee ....1<br>Dee'ni ....2<br>Dibuuxoomma/o........... 8	Ee ....1<br>Dee'ni ....2<br>Dibuuxoomma/o............ 8	Ee ....1<br>Dee'ni ....2<br>Dibuuxoomma/o............ 8	Sai X 210

Q209	Ki'ne giddo agobere ankarro horoonsi'rinohu ayeti?	Ayimmate Kaarde<br>5. __________<br>6. __________<br>7. _________<br>8. ________	Ayimmate Kaarde<br>5. __________<br>6. __________<br>7. _________<br>8. _________	Ayimmate Kaarde<br>1__________<br>2__________<br>3_________<br>4_________	
Q210	Agobere horoonsidhinoonnikkihu mayraati?	Shekkeere nookkihuraati……1<br>Shekkeere tareessitanno biinnicho nookkihuraati… 2<br>Agobere wodhineemmo dargi noonkekkihuraati …3<br>Agoberete fooli biso darshiishannohuraati ………………4<br>Agobere iimaanni wodhummaro/moro hunkiishshaae /foole tayissannohuraati.. 5<br>Agobere horoonsi'ra mitiinsitannohuraati ….6<br>Agoberete suudi injaannokkihuraati..7<br>Mine agobere afi'noommokkihuraati …..8<br>Wole xawisi ……9<br>Diafoommo/a<br>……....98	Shekkeere nookkihuraati……1<br>Shekkeere tareessitanno biinnicho nookkihuraati---- 2<br>wodhineemmo dargi noonkekkihuraati …3<br>Agoberete fooli biso darshiishannohuraati ………………4<br>Agobere iimaanni wodhummaro/moro hunkiishshaae /foole tayissannohuraati.. 5<br>Agobere horoonsi'ra mitiinsitannohuraati ….6<br>Agoberete suudi injaannokkihuraati..7<br>Mine agobere afi'noommokkihuraati …..8<br>Wole xawisi ……9<br>Diafoommo/a<br>……....98	Shekkeere nookkihuraati……1<br>Shekkeere tareessitanno biinnicho nookkihuraati… 2<br>wodhineemmo dargi noonkekkihuraati --…3<br>Agoberete fooli biso darshiishannohuraati ………………4<br>Agobere iimaanni wodhummaro/moro hunkiishshaae /foole tayissannohuraati.5<br>Agobere horoonsi'ra mitiinsitannohuraati ….6<br>Agoberete suudi injaannokkihuraati.7<br>Mine agobere afi'noommokkihuraati …..8<br>Wole xawisi ……9<br>Diafoommo/a<br>……....98	
Q211	Woradu fayyimmate biro biinne gargartanno xagga mini'ne qarqarira kiiffe egentino'ne?	Ee …………….1<br>Dee'ni ..…………...2<br>Dibuuxoommo/a..………8			Sai X215
Q212	Meu again albaanni xagga kiinfoonni'ne?<br>*(xagga kiinfoonni agana wo'mitinokkiro ''0'' borreessi)*	Agannate albaanni [___/___]<br>Dibuunxoonni …..8			
Q213	Saihu 12 agani giddo galtinanni minira kuula/pilaastere xallinoonni?	Ee ....1<br>Dee'ni.. 2			
Q214	Meu agani albanni girgiddaho pilaasitere/kuula xallinoonni?	Agannate albaanni, _____ _____			

	(agana wo’mitinokkiro ‘’0’’ borreessi)		
