# Supplementary material for: A decline in the coverage and utilization of long-lasting insecticidal nets in Southern Ethiopia: A repeated cross-sectional study
Source: PLoS One. 2025 Apr 24;20(4):e0322342. doi: 10.1371/journal.pone.0322342 (PMC12021140; doi:10.1371/journal.pone.0322342)
Supplement: S5 File — Models of second survey and additional variables. (DOCX) [file pone.0322342.s005.docx]

**Predictors of sufficiency of one LLIN for every two people, Sidama, 2023**

**Second survey**

| Variable | Categories | Household met one LLIN to two members | | Logistic regression | | | | Multilevel logistic regression | |
| --- | --- | --- | --- | --- | --- | --- | --- | --- | --- |
|  |  | No  No (%) | Yes  No (%) | COR (95%CI) | P-value | AOR (95%CI) | P-value | AOR (95%CI) | P-value |
| Community level |  |  |  |  |  |  |  |  |  |
| Districts | Boricha | 399 (55.4) | 321 (45.6) | 1 |  | 1 |  | 1 |  |
|  | Bilate Zuria | 745 (84.4) | 138 (15.6) | 0.23 [0.18-0.29] | <0.001 | 0.20 [0.16-0.26] | <0.001 | 0.12 [0.06-0.25] | **<0.001** |
| Household level |  |  |  |  |  |  |  |  |  |
| Sex of head | Male | 1024 (72.2) | 395 (27.8) | 1 |  | 1 |  | 1 |  |
|  | Female | 120 (65.2) | 64 (34.8) | 1.38 [1.00-1.91] | 0.05 | 1.84 [1.23-2.64] | 0.001 | 2.43 [1.60-3.67] | **<0.001** |
| Age of head | Age of head | 1144 (71.4) | 459 (28.6) | 1.00 [0.99-1.00] | 0.34 | 0.99 [0.98-1.00] | 0.17 | 0.99 [0.98-1.01] | 0.45 |
| Education of head | Education of head | 1144 (71.4) | 459 (28.6) | 1.13 [1.03-1.24] | 0.01 | 1.15 [1.02-1.29] | 0.02 | 1.22 [1.06-1.39] | **0.004** |
| Occupation of head | Farmer/Housewife | 839 (73.0) | 311 (27.0) | 1 |  | 1 |  | 1 |  |
|  | Others | 305 (67.3) | 148 (32.7) | 1.31 [1.03-1.66] | 0.03 | 1.28 [0.97-1.69] | 0.08 | 1.07 [0.78-1.47] | 0.68 |
| Wealth index | Wealth index | 1144 (71.4) | 459 (28.6) | 0.98 [0.91-1.06] | 0.58 | 0.90 [0.83-0.98] | 0.02 | 0.95 [0.86-1.04] | 0.25 |

**Model summary**

| Dependent variable | Round | Model | Parameter | | | | | | |
| --- | --- | --- | --- | --- | --- | --- | --- | --- | --- |
|  |  |  | ICC | Intercept | Cluster variance | Wald Chi-square test | Likelihood-ratio (LR) test) | AIC | BIC |
| LLIN sufficiency | First survey | Null | 0.37 | 0.43 | 1.96 | Reference | 339.81, P<0.001 | 1812.00 | 1822.81 |
|  |  | Fitted | 0.26 | 1.40 | 1.14 | 62.96, P<0.001 | 186.56, P<0.001 | 1762.69 | 1805.94 |
|  | Second survey | Null | 0.43 | 0.26 | 2.48 | Reference | 325.47, P<0.001 | 1598.41 | 1609.17 |
|  |  | Fitted | 0.31 | 0.68 | 1.46 | 54.34, P<0.001 | 184.08, P<0.001 | 1557.79 | 1600.83 |

**Predictors of LLIN use among individuals, Sidama, 2023**

**Second survey**

| Variable | Category | Member used LLIN | | Logistic regression | | | | Multilevel logistic regression | |
| --- | --- | --- | --- | --- | --- | --- | --- | --- | --- |
|  |  | No  No (%) | Yes  No (%) | COR (95%CI) | P-value | AOR (95%CI) | P-value | AOR (95%CI) | P-value |
| Community level |  |  |  |  |  |  |  |  |  |
| Districts | Boricha | 2326 (73.4) | 845 (26.6) | 1 |  | 1 |  | 1 |  |
|  | Bilate Zuria | 3511 (85.4) | 602 (14.6) | 0.47 [0.42-0.53] | <0.001 | 0.48 [0.42-0.54] | <0.001 | 0.57 [0.27-1.22] | 0.15 |
| Household level |  |  |  |  |  |  |  |  |  |
| Education of head | Education of head | 5837 (80.1) | 1447 (19.9) | 1.15 [1.09-1.21] | <0.001 | 1.12 [1.06-1.18] | <0.001 | 1.33 [1.24-1.42] | <0.001 |
| Occupation of head | Farmer/Housewife | 4331 (81.5) | 983 (18.5) | 1 |  | 1 |  | 1 |  |
|  | Others | 1506 (76.5) | 464 (23.5) | 1.14 [1.20-1.54] | <0.001 | 1.27 [1.11-1.46] | 0.001 | 0.95 [0.80-1.13] | 0.58 |
| Family size | Family size | 5837 (80.1) | 1447 (19.9) | 0.82 [0.79-0.85] | <0.001 | 0.86 [0.83-0.89] | <0.001 | 0.85 [0.81-0.89] | <0.001 |
| Wealth index | Wealth index | 5837 (80.1) | 1447 (19.9) | 0.98 [0.94-1.02] | 0.37 | 0.94 [0.90-0.98] | 0.01 | 0.99 [0.94-1.04] | 0.67 |
| Individual level |  |  |  |  |  |  |  |  |  |
| Sex | Male | 2877 (79.7) | 735 (20.3) | 1 |  | 1 |  | 1 |  |
|  | Female | 2960 (80.6) | 712 (19.4) | 0.94 [0.84-1.06] | 0.31 | 0.93 [0.83-1.05] | 0.27 | 0.91 [0.79-1.05] | 0.17 |
| Age of head | < 5 | 532 (81.0) | 125 (19.0) | 1 |  | 1 |  | 1 |  |
|  | 5-14 | 1603 (89.6) | 187 (10.4) | 0.49 [0.39-0.63] | <0.001 | 0.60 [0.47-0.77] | <0.001 | 0.60 [0.45-0.80] | 0.001 |
|  | 15+ | 3702 (76.5) | 1135 (23.5) | 1.30 [1.06-1.60] | 0.11 | 1.52 [1.26-1.88] | <0.001 | 2.04 [1.59-2.61] | <0.001 |

**Model summary**

| Dependent variable | Round | Model | Parameter | | | | | | |
| --- | --- | --- | --- | --- | --- | --- | --- | --- | --- |
|  |  |  | ICC | Intercept | Variance | Wald Chi-square test | Likelihood-ratio (LR) test) | AIC | BIC |
| LLIN use | First survey | Null | 0.16 | 0.40 | 0.61 | Reference | 684.36, P<0.001 | 9229.24 | 9243.23 |
|  |  | Fitted | 0.18 | 0.18 | 0.73 | 642.71, P<0.001 | 738.84, P<0.001 | 8476.54 | 8546.48 |
|  | Second survey | Null | 0.36 | 0.16 | 1.85 | Reference | 1516.73, P<0.001 | 5749.88 | 5763.67 |
|  |  | Fitted | 0.37 | 0.21 | 1.97 | 297.53, P<0.001 | 1421.77, P<0.001 | 5427.03 | 5495.96 |

**Predictors of starting LLIN use among individuals, Sidama, 2023**

| Variable | Category | Member started using LLIN | | Logistic regression | | | | Multilevel logistic regression | |
| --- | --- | --- | --- | --- | --- | --- | --- | --- | --- |
|  |  | No  No (%) | Yes  No (%) | COR (95%CI) | P-value | AOR (95%CI) | P-value | AOR (95%CI) | P-value |
| Community level |  |  |  |  |  |  |  |  |  |
| Districts | Boricha | 1709 (78.4) | 472 (21.6) | 1 |  | 1 |  | 1 |  |
|  | Bilate Zuria | 2519 (89.2) | 304 (10.8) | 0.44 [0.37-0.51] | <0.001 | 0.45 [0.38-0.53] | <0.001 | 0.52 [0.21-1.24] | 0.14 |
| Household level |  |  |  |  |  |  |  |  |  |
| Education of head | Education of head | 4228 (84.5) | 776 (15.5) | 1.17 [1.09-1.25] | <0.001 | 1.12 [1.04-1.21] | 0.002 | 1.32 [1.20-1.45] | <0.001 |
| Occupation of head | Farmer/Housewife | 3189 (86.2) | 510 (13.8) | 1 |  | 1 |  | 1 |  |
|  | Others | 1039 (79.6) | 266 (20.4) | 1.60 [1.36-1.88] | <0.001 | 1.51 [1.26-1.81] | <0.001 | 1.05 [0.83-1.33] | 0.66 |
| Family size | Family size | 4228 (84.5) | 776 (15.5) | 0.81 [0.77-0.85] | <0.001 | 0.86 [0.82-0.91] | <0.001 | 0.83 [0.78-0.89] | <0.001 |
| Wealth index | Wealth index | 4228 (84.5) | 776 (15.5) | 0.98 [0.92-1.03] | 0.39 | 0.92 [0.87-0.98] | 0.005 | 0.98 [0.91-1.05] | 0.56 |
| Individual level |  |  |  |  |  |  |  |  |  |
| Sex | Male | 2050 (87.3) | 299 (12.7) | 1 |  | 1 |  | 1 |  |
|  | Female | 2178 (82.0) | 477 (18.0) | 1.50 [1.28-1.76] | <0.001 | 1.46 [1.24-1.72] | <0.001 | 1.34 [1.10-1.63] | <0.001 |
| Age of head | < 5 | 451 (81.8) | 100 (18.2) | 1 |  | 1 |  | 1 |  |
|  | 5-14 | 1366 (91.4) | 128 (8.6) | 0.42 [0.32-0.56] | <0.001 | 0.52 [0.39-0.69] | <0.001 | 0.55 [0.39-0.78] | 0.001 |
|  | 15+ | 2411 (81.5) | 548 (18.5) | 1.02 [0.81-1.30] | 0.84 | 1.21 [0.95-1.56] | 0.12 | 1.87 [1.38-2.53] | <0.001 |

**Model summary**

| Dependent variable | Model | Parameter | | | | | | |
| --- | --- | --- | --- | --- | --- | --- | --- | --- |
|  |  | ICC | Intercept | Variance | Wald Chi-square test | Likelihood-ratio (LR) test) | AIC | BIC |
| Decline in LLIN use | Null | 0.43 | 0.10 | 2.48 | Reference | 1083.38, P<0.001 | 3238.21 | 3251.24 |
|  | Fitted | 0.44 | 0.15 | 2.55 | 180.05, P<0.001 | 989.64, P<0.001 | 3048.49 | 3113.67 |

**Predictors of consistent LLIN use among individuals, Sidama, 2023**

| Variable | Category | Member used LLIN during both surveys | | Logistic regression | | | | Multilevel logistic regression | |
| --- | --- | --- | --- | --- | --- | --- | --- | --- | --- |
|  |  | No  No (%) | Yes  No (%) | COR (95%CI) | P-value | AOR (95%CI) | P-value | AOR (95%CI) | P-value |
| Community level |  |  |  |  |  |  |  |  |  |
| Districts | Boricha | 2798 (88.2) | 373 (11.8) | 1 |  | 1 |  | 1 |  |
|  | Bilate Zuria | 3815 (92.7) | 298 (7.3) | 0.58 [0.50-0.69] | <0.001 | 0.61 [0.51-0.72] | <0.001 | 0.75 [0.37-1.50] | 0.42 |
| Household level |  |  |  |  |  |  |  |  |  |
| Education of head | Education of head | 6613 (90.8) | 671 (9.2) | 1.20 [1.12-1.28] | <0.001 | 1.21 [1.13-1.30] | <0.001 | 1.36 [1.25-1.48] | <0.001 |
| Occupation of head | Farmer/Housewife | 4841 (91.1) | 473 (8.9) | 1 |  | 1 |  | 1 |  |
|  | Others | 1772 (89.9) | 198 (10.1) | 1.14 [0.96 -1.36] | 0.13 | 1.00 [0.82-1.22] | 0.99 | 0.84 [0.68-1.04] | 0.11 |
| Family size | Family size | 6613 (90.8) | 671 (9.2) | 0.79 [0.76-0.84] | <0.001 | 0.82 [0.78-0.87] | <0.001 | 0.83 [0.78-0.88] | <0.001 |
| Wealth index | Wealth index | 6613 (90.8) | 671 (9.2) | 0.96 [0.91-1.02] | 0.17 | 0.94 [0.89-1.00] | 0.06 | 1.00 [0.93-1.06] | 0.95 |
| Individual level |  |  |  |  |  |  |  |  |  |
| Sex | Male | 3176 (87.9) | 436 (12.1) | 1 |  | 1 |  | 1 |  |
|  | Female | 3437 (93.6) | 235 (6.4) | 0.50 [0.42-0.59] | <0.001 | 0.47 [0.40-0.56] | <0.001 | 0.43 [0.36-0.52] | <0.001 |
| Age of head | < 5 | 632 (96.2) | 25 (3.8) | 1 |  | 1 |  | 1 |  |
|  | 5-14 | 1731 (96.7) | 59 (3.3) | 0.86 [0.53-1.39] | 0.54 | 1.12 [0.69-1.82] | 0.64 | 1.16 [0.71-1.91] | 0.55 |
|  | 15+ | 4250 (87.9) | 587 (12.1) | 3.49 [2.32-5.26] | <0.001 | 4.43 [2.92-6.71] | <0.001 | 5.36 [3.49-8.22] | <0.001 |

**Model summary**

| Dependent variable | Model | Parameter | | | | | | |
| --- | --- | --- | --- | --- | --- | --- | --- | --- |
|  |  | ICC | Intercept | Variance | Wald Chi-square test | Likelihood-ratio (LR) test) | AIC | BIC |
| Decline in LLIN use | Null | 0.29 | 0.06 | 1.36 | Reference | 446.21, P<0.001 | 4036.20 | 4049.99 |
|  | Fitted | 0.32 | 0.04 | 1.51 | 307.98, P<0.001 | 442.02, P<0.001 | 3670.06 | 3738.99 |

**Predictors of decline in LLIN use among individuals, Sidama, 2023**

| Variable | Category | Member stopped using LLIN | | Logistic regression | | | | Multilevel logistic regression | |
| --- | --- | --- | --- | --- | --- | --- | --- | --- | --- |
|  |  | No  No (%) | Yes  No (%) | COR (95%CI) | P-value | AOR (95%CI) | P-value | AOR (95%CI) | P-value |
| Community level |  |  |  |  |  |  |  |  |  |
| Districts | Boricha | 373 (37.7) | 617 (62.3) | 1 |  | 1 |  | 1 |  |
|  | Bilate Zuria | 298 (23.1) | 992 (76.9) | 2.01 [1.68-2.41] | <0.001 | 1.97 [1.63-2.37] | <0.001 | 1.66 [0.80-3.47] | 0.17 |
| Household level |  |  |  |  |  |  |  |  |  |
| Education of head | Education of head | 671 (29.4) | 1609 (70.6) | 0.97 [0.90-1.05] | 0.42 |  |  |  |  |
| Occupation of head | Farmer/Housewife | 473 (29.3) | 1142 (70.7) | 1 |  |  |  |  |  |
|  | Others | 198 (29.8) | 467 (70.2) | 0.98 [0.80-1.19] | 0.82 |  |  |  |  |
| Family size | Family size | 671 (29.4) | 1609 (70.6) | 1.14 [1.08-1.20] | <0.001 | 1.13 [1.06-1.20] | <0.001 | 1.13 [1.06-1.20] | <0.001 |
| Wealth index | Wealth index | 671 (29.4) | 1609 (70.6) | 0.98 [0.92-1.04] | 0.51 | 1.02 [0.94-1.10] | 0.67 | 1.02 [0.94-1.10] | 0.67 |
| Individual level |  |  |  |  |  |  |  |  |  |
| Sex | Male | 436 (34.5) | 827 (65.5) | 1 |  | 1 |  | 1 |  |
|  | Female | 235 (23.1) | 782 (76.9) | 1.75 [1.45-2.11] | <0.001 | 1.73 [1.43-2.09] | <0.001 | 1.63 [1.31-2.02] | <0.001 |
| Age of head | < 5 | 25 (23.6) | 81 (76.4) | 1 |  | 1 |  | 1 |  |
|  | 5-14 | 59 (20.0) | 237 (80.0) | 1.24 [0.73-2.11] | 0.43 | 1.17 [0.68-2.02] | 0.57 | 1.48 [0.82-2.69] | 0.19 |
|  | 15+ | 587 (31.3) | 1291 (68.7) | 0.68 [0.43-1.07] | 0.10 | 0.67 [0.42-1.07] | 0.10 | 0.70 [0.42-1.19] | 0.19 |

**Model summary**

| Dependent variable | Model | Parameter | | | | | | |
| --- | --- | --- | --- | --- | --- | --- | --- | --- |
|  |  | ICC | Intercept | Variance | Wald Chi-square test | Likelihood-ratio (LR) test) | AIC | BIC |
| Decline in LLIN use | Null | 0.35 | 3.28 | 1.77 | Reference | 393.44, P<0.001 | 2373.72 | 2385.19 |
|  | Fitted | 0.33 | 1.65 | 1.65 | 51.81, P<0.001 | 323.34, P<0.001 | 2330.65 | 2376.50 |
